# Advances in nanomaterials for treatment of hypoxic tumor

**DOI:** 10.1093/nsr/nwaa160

**Published:** 2020-07-08

**Authors:** Mei-Zhen Zou, Wen-Long Liu, Han-Shi Chen, Xue-Feng Bai, Fan Gao, Jing-Jie Ye, Han Cheng, Xian-Zheng Zhang

**Affiliations:** The Institute for Advanced Studies, Key Laboratory of Biomedical Polymers of Ministry of Education & Department of Chemistry, Wuhan University, Wuhan 430072, China; School of Chemistry and Materials Science, South-Central University for Nationalities, Wuhan 430074, China; The Institute for Advanced Studies, Key Laboratory of Biomedical Polymers of Ministry of Education & Department of Chemistry, Wuhan University, Wuhan 430072, China; The Institute for Advanced Studies, Key Laboratory of Biomedical Polymers of Ministry of Education & Department of Chemistry, Wuhan University, Wuhan 430072, China; The Institute for Advanced Studies, Key Laboratory of Biomedical Polymers of Ministry of Education & Department of Chemistry, Wuhan University, Wuhan 430072, China; The Institute for Advanced Studies, Key Laboratory of Biomedical Polymers of Ministry of Education & Department of Chemistry, Wuhan University, Wuhan 430072, China; The Institute for Advanced Studies, Key Laboratory of Biomedical Polymers of Ministry of Education & Department of Chemistry, Wuhan University, Wuhan 430072, China; The Institute for Advanced Studies, Key Laboratory of Biomedical Polymers of Ministry of Education & Department of Chemistry, Wuhan University, Wuhan 430072, China

**Keywords:** nanomaterials, hypoxic tumor, tumor therapy, oxygen

## Abstract

The hypoxic tumor microenvironment is characterized by disordered vasculature and rapid proliferation of tumors, resulting from tumor invasion, progression and metastasis. The hypoxic conditions restrict efficiency of tumor therapies, such as chemotherapy, radiotherapy, phototherapy and immunotherapy, leading to serious results of tumor recurrence and high mortality. Recently, research has concentrated on developing functional nanomaterials to treat hypoxic tumors. In this review, we categorize such nanomaterials into (i) nanomaterials that elevate oxygen levels in tumors for enhanced oxygen-dependent tumor therapy and (ii) nanomaterials with diminished oxygen dependence for hypoxic tumor therapy. To elevate oxygen levels in tumors, oxygen-carrying nanomaterials, oxygen-generating nanomaterials and oxygen-economizing nanomaterials can be used. To diminish oxygen dependence of nanomaterials for hypoxic tumor therapy, therapeutic gas-generating nanomaterials and radical-generating nanomaterials can be used. The biocompatibility and therapeutic efficacy of these nanomaterials are discussed.

## INTRODUCTION

Hypoxia is a characteristic of most tumors [1,2]. Tumor hypoxia results from consumption of large amounts of oxygen by tumor cells for rapid proliferation, while the tumor vasculature is malformed and abnormal, limiting the adequate supply of oxygen [3,4]. In addition, the high interstitial pressure limits oxygen diffusion into deep tumor layers, leading to extreme hypoxia in deep tumors [5,6]. Compared with oxygenated healthy tissues, hypoxic tumors often display, among other things, high levels of reactive oxygen species (ROS), low pH and altered metabolism [7]. Hypoxia generally leads to intratumoral heterogeneity and inhibition of innate and adaptive immune responses, which promotes the probability of tumor metastasis [8,9]. Furthermore, it is recognized that some tumor cells can survive in hypoxic conditions [10], and even worse, the hypoxia-tolerant tumor cells are more resistant to traditional tumor therapies including radiotherapy [11,12], chemotherapy [13] and photodynamic therapy (PDT) [14,15]. Recently, several trials have been performed aiming to improve the hypoxic microenvironment, e.g. through use of inspiratory hyperoxia. Unfortunately, it is difficult to apply this technique to clinical therapy because of the severe structural malformation of microvessels in tumors and the side effects of hyperbaric oxygen therapy [16]. Thus, it is critically important to develop reliable methods for treatment of hypoxic tumors.

Nanomaterials have developed rapidly over the past decade, opening up new areas in biomedical applications including bioimaging, targeted drug delivery and tumor therapy [17–20]. As a result of the enhanced permeation and retention (EPR) effect, nanomaterials have prior accumulation in tumor tissues [21]. Also, nanomaterials are equipped to carry and deliver drugs (small molecules, proteins, DNA, etc.) via surface attachment, encapsulation and entrapping [22–24]. Nanomaterials serving as drug delivery systems have many advantages: (i) nanomaterials are conducive to change the pharmacokinetics of drugs and increase delivery efficiency [25]; (ii) nanomaterials possess high drug encapsulation capacity and controllable drug delivery in the circulation process to avoid drug leakage [26]; (iii) nanomaterials can be modified easily and further enhance multifunctionality, solubility and stability of nanomedicine, to make it favorable for *in vivo* administration [27].

**Figure 1.**
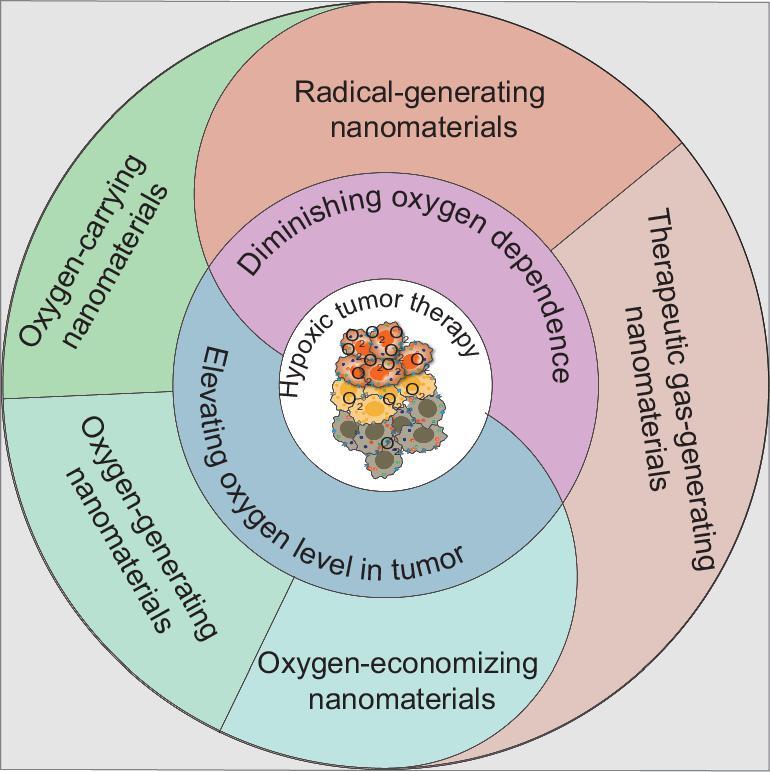
Strategies for treatment of hypoxic tumor with nanomaterials.

Here, we summarize recent studies on nanomaterials in hypoxic tumor therapy (Fig. 1). It is recognized that the hypoxic tumor microenvironment is attributed to the larger consumption of oxygen than the supply of oxygen in tumor tissue. Thus, elevating oxygen levels in tumor tissues would be the most direct way to treat hypoxic tumors. Diminishing oxygen dependence for hypoxic tumor therapy has also been postulated as a therapeutic option in recent years, e.g. by taking advantage of penetration of therapeutic gas or generation of toxic substances *in situ* in hypoxic tumor. In this review, two main aspects are discussed regarding use of nanomaterials to treat hypoxic tumors: (i) elevating oxygen level at tumor sites with use of nanomaterials including oxygen-carrying nanomaterials, oxygen-generating nanomaterials and oxygen-economizing nanomaterials, and (ii) diminishing oxygen dependence of nanomaterials by use of therapeutic gas-generating nanomaterials and radical-generating nanomaterials (Table 1). Also, potential challenges, including biocompatibility, and future prospects for nanomaterials to treat hypoxic tumors are discussed.

**Table 1.** Summary of nanomaterials for treatment of hypoxic tumor.

Strategies	Nanomaterials for treatment of hypoxic tumor	Examples	Key references
Elevating oxygen level in tumor	Oxygen-carrying nanomaterials	Oxygen nanobubbles Hb-containing oxygen-carrying nanomaterials	[35–40] [45–54]
		PFC-containing oxygen-carrying nanomaterials	[59–67]
	Oxygen-generating nanomaterials	H_2_O_2_-based oxygen-generating nanomaterials	[69–75,84–87,89–91,95–102]
		H_2_O-based oxygen-generating nanomaterials	[103–106]
	Oxygen-economizing nanomaterials	Respiration-inhibiting oxygen-economizing nanomaterials	[109–112,114–116]
Diminishing oxygen dependence	Therapeutic gas-generating nanomaterials	NO-generating nanomaterials**·**OH-generating nanomaterials	[126,127][138–143]
	Radical-generating nanomaterials	**·**R-generating nanomaterials	[146–148]

## USE OF NANOMATERIALS TO ELEVATE OXYGEN LEVELS IN HYPOXIC TUMOR THERAPY

Many strategies to treat tumors are oxygen-dependent, including chemotherapy [28], radiotherapy [29,30], PDT [14] as well as immunotherapy [9,31]. Through various mechanisms, the low oxygen concentration in tumor tissues limits the efficacy of these therapies. To solve these problems, approaches have been made to modulate the concentration of oxygen in tumors. Both oxygen-carrying nanomaterials and oxygen-generating nanomaterials provide oxygen to relieve the hypoxic microenvironment and enhance the therapeutic effects of oxygen-dependent therapies. In addition, oxygen content can be temporarily improved by inhibiting respiration to economize oxygen in tumor cells.

### Oxygen-carrying nanomaterials for hypoxic tumor therapy

Nanobubbles consisting of a stabilizing monolayered shell and gas core have the potential to carry gas molecules (e.g. oxygen) [32,33]. Oxygen nanobubbles with the ability to carry oxygen have been explored in delivery of oxygen for tumor oxygenation and enhancement of tumor therapy [34,35]. Such oxygen nanobubbles were constructed with an oxygen core and nanolayered shell including lipid, polymer, dextran and gas vesicles [36,37]. Drugs can be loaded within the nanobubbles either by encapsulation in the core or by coating the outer shell in a covalent or non-covalent method according to the hydrophobic or hydrophilic properties of the drugs. Oxygen nanobubbles have been used to suppress expression of hypoxic inducible factor-1α (HIF-1α) to enhance the therapeutic effects of radiotherapy and chemotherapy when mediated with different treatments [38,39]. Furthermore, dependent on external stimuli, oxygen may responsive-release in tumors to avoid premature oxygen release and reduce side effects. Song *et al*. used acetylated dextran, a pH-responsive polymer, to serve as the shell of oxygen nanobubbles, thus enabling release of oxygen to alleviate tumor hypoxia in the tumor microenvironment [34]. Although progress has been made with oxygen nanobubbles to enhance hypoxic tumor therapy, there remain problems with stability and storage of oxygen nanobubbles to avoid premature oxygen leakage [40].

Hemoglobin (Hb), an abundant natural metalloprotein in red blood cells (RBCs), is mainly used to transport and deliver oxygen in mammals. It is constructed of four globular polypeptide submits (α1, β1, α2, β2), each of which is composed of a structure with a porphyrin ring. The center of the porphyrin ring contains a ferrous ion (Fe^2+^), which is responsible for oxygen transport of Hb and each Fe^2+^ binds one oxygen molecule [41]. As an oxygen transport protein, Hb has drawn the interest of researchers towards blood substitutes. However, the free Hb cannot be directly used to transport oxygen because it can be easily broken into dimers with a short circulation time, high organ toxicity and strong oxygen affinity to obstruct oxygen release [42,43]. As a consequence, modification or encapsulation of Hb to prevent its breakage is necessary for effective oxygen transport *in vivo*.

**Figure 2.**
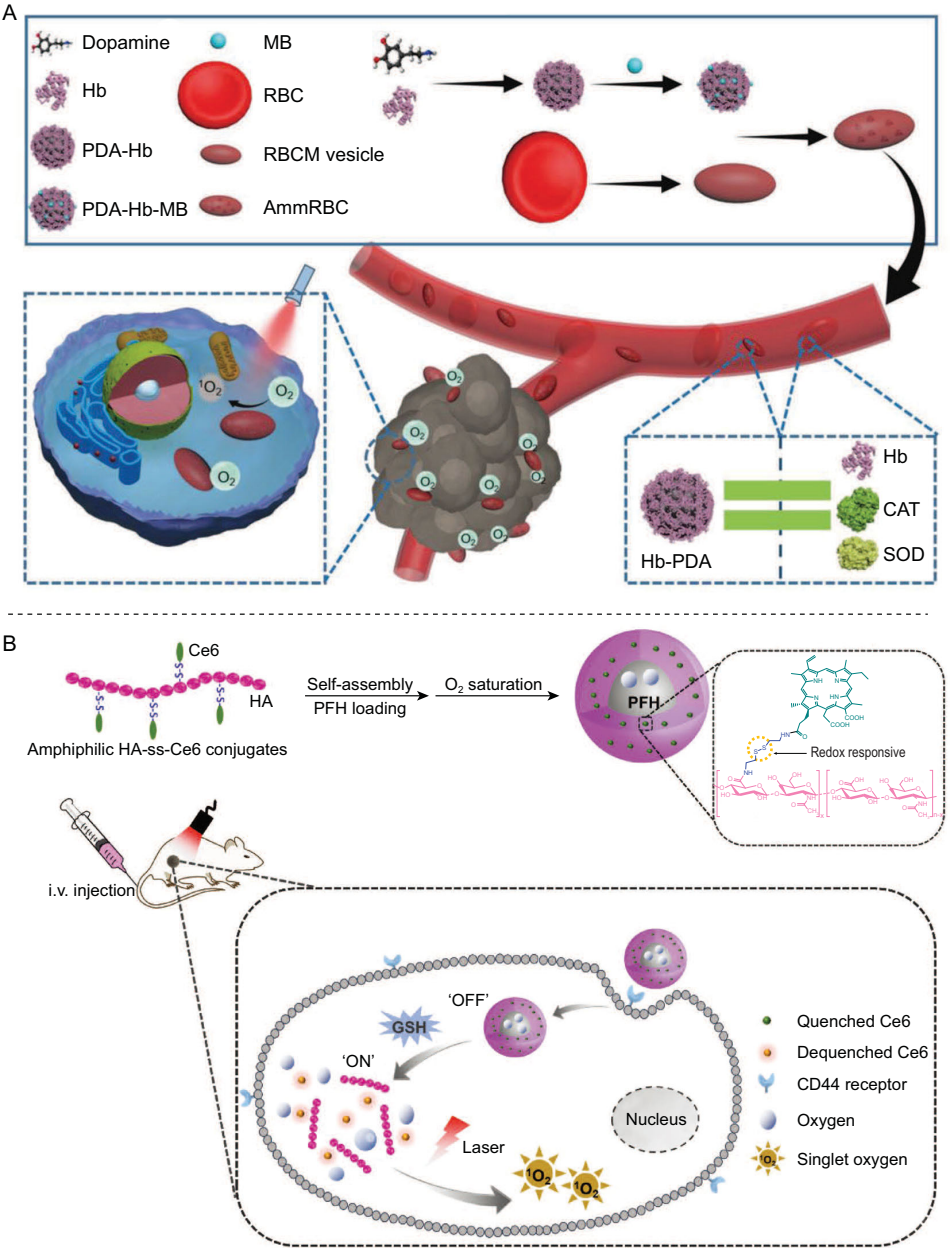
Oxygen-carrying nanomaterials to enhance tumor therapy. (A) Aggressive man-made RBCs containing polydopamine which functions like CAT and SOD in RBCs to protect Hb from oxidant damage during the circulation for hypoxia-resistant PDT. Reproduced with permission from [54]. Copyright 2018, Wiley-VCH. (B) Design and functions of redox-responsive and oxygen-delivering tumor-targeted photosensitizer to enhance the efficacy of tumor PDT. Reproduced with permission from [64]. Copyright 2019, Wiley-VCH.

As the source of Hb, each RBC approximately contains 270 million Hb molecules [44]. Thus, RBCs have been directly used to transport oxygen for alleviation of hypoxia. Oxygen can be released from Hb in RBCs and diffuses to the surroundings, where it is then rapidly consumed by tumor cells closed to capillaries. When RBCs were used to carry a photosensitizer, generation of ROS was enhanced by adjacent Hb-carried oxygen [45]. As tumor vessels are abnormal and they leak, it is difficult for RBCs of larger size to infiltrate into deep tumors. Because of the EPR effects of nanomaterials, researchers have hybridized Hb with proteins or polymers at the nanoscale to enhance penetration and accumulation of Hb in tumor tissues [46,47]. Nanomaterials containing human serum albumin and Hb (C@HOPC) were prepared via a protein hybridization approach as oxygen nanocarriers for amplified PDT. C@HOPC showed gradual release of oxygen in hypoxic solution for many minutes [46]. In addition, encapsulation of Hb in poly lactic-co-glycolic acid (PLGA) or liposomes constructed as nanoscale oxygen carriers, have been used to reverse hypoxia-induced chemoresistance when combined with chemotherapeutic drugs [28,48]. However, Hb is very susceptible to oxide, resulting in a partial loss of oxygen-transporting capacity and even the generation of toxic substances during circulation *in vivo*. These side effects were exacerbated when Hb-based nanoparticles encountered the pathological abundant oxidizing hydrogen peroxide (H_2_O_2_) in the tumor environment [49]. To reduce oxidative damage to Hb, RBCs possess their own antioxidation system including glutathione (GSH) and antioxidant enzymes, such as catalase (CAT) and superoxide dismutase (SOD) [50,51]. To protect Hb from oxidation, work has been done to simulate construction of RBCs with an anti-oxidative system. In 1998, SOD and CAT were used to crosslink Hb with crosslinker glutaraldehyde to prevent Hb from disassociation and oxidation [52]. Other alternative antioxidant enzymes, such as rubrerythrin, have been used to protect Hb against high oxidative level [53]. Although these methods protect Hb from generating toxic substances, the crosslinker glutaraldehyde is a biocide, and is toxic to organisms, thus limiting the biomedical applications of crosslinked Hb. Considering this problem, Zhang's group designed a man-made RBC with enzyme-mimicking polydopamine, which could simulate the functions of CAT and SOD. The man-made RBC prevented Hb from oxidative destruction during blood circulation and in the tumor tissues. When methylene blue (MB) was absorbed in the man-made RBC, oxygen which was carried by the protected Hb relieved tumor hypoxia, and generation of ROS by MB enhanced tumor PDT under light irradiation (Fig. 2A). This method, based on nanomaterials, has potential in application of isolated Hb from blood, so as to address the shortage of packed RBCs in clinics [54].

Hb has good biocompatibility, but its oxygen-transporting efficiency is limited by its binding sites as each Hb can reversibly bind and release only four oxygen molecules. Benefiting from extremely low polarizability of fluorine, perfluorocarbon (PFC) possesses great gas solubility. Liquid PFC, as a nearly ideal and gas-like fluid, can easily dissolve a substance with similar low cohesive force, such as oxygen, carbon dioxide (CO_2_), nitrogen (N_2_), nitric oxide (NO), etc. According to clinical data, 1.5 times more Hb is required than PFC to carry the same content of oxygen [55]. In clinic, PFC emulsions (e.g. Fluosol-DA-20%) have been approved for intravenous use by the United States Food and Drug Administration (FDA) as an artificial blood substitute [56]. Because of its biocompatibility, PFC compounds have also been used as contrast agents for magnetic resonance imaging (MRI) and ultrasound (US) to diagnose diseases [57,58], and its oxygen-carrying capacity means that application of PFC to relieve tumor hypoxia has been considered in recent years [59]. Lipid-coated perfluorohexane and photosensitizer can improve the efficacy of PDT. The cytotoxicity of the nanomaterials with NIR irradiation (about 87%) to CT26 in hypoxia was much higher than that of lipid-coated photosensitizer (about 7%) *in vitro*. Although PFC can carry large amounts of oxygen, its super-hydrophobic structure makes it hard to modify, limiting its development in tumor therapy. Even so, many researchers have considered remolding PFC to carry oxygen to tumor tissues. Firstly, with their good loading ability, hollow nanoparticles including hollow Bi_2_Se_3_ and hollow mesoporous silica nanoparticles were used to load PFC. Hollow Bi_2_Se_3_ nanoparticles were able to simultaneously absorb X-ray irradiation and near-infrared ray (NIR) light. PFC saturated with oxygen in hollow Bi_2_Se_3_ can increase oxygen concentrations sharply in hypoxic solutions within about 2 minutes. The released oxygen could overcome hypoxia-associated RT-resistance of tumor and alleviate DNA damage in the RT process [60]. Hollow mesoporous silica nanoparticles were not only loaded with PFC to oxygenate tumor, but also loaded with photosensitizer (Ce6) or sonosensitizers (IR780) to generate ROS under NIR light or ultrasound [61,62]. PFC can also self-assemble with proteins or polymers (e.g. human serum albumin, hyaluronic acid (HA) and PLGA) to fabricate nanoparticles for enhancement of tumor therapy [63]. For example, redox-responsive amphiphilic HA-ss-Ce6 conjugates were self-assembled with PFC to construct tumor-targeted nanoparticles. The nanoparticles would accumulate in tumor sites through the EPR effect and enter the tumor cells via HA-related tumor overexpression receptor CD44 mediated endocytosis. Then the nanoparticles dissociate in the tumor redox microenvironment accompanied by fluorescence recovery and delivery of PFC with oxygen, thus enhancing generation of ROS for tumor therapy (Fig. 2B) [64]. Moreover, RBC membranes could be introduced to emulsify PFC to construct artificial RBCs for oxygen transportation [65]. In addition, artificial natural killer cells have been designed with a PFC core to carry oxygen for alleviating the hypoxic microenvironment. The artificial natural killer cells with the carried oxygen could selectively kill tumor cells and polarize macrophages by generation of H_2_O_2_ under glucose oxidase [66]. The encapsulation or emulsification of PFC could gradually deliver oxygen in tumor tissues, and the oxygen could also be rapidly released under the above-mentioned NIR light and ultrasound treatment, demonstrating that controllable release of oxygen could be realized in tumor therapy [55,67]. Thus, the therapeutic effects of oxygen-dependent tumor therapy methods could be enhanced by PFC-associated nanomaterials through transport of oxygen.

Compared with Hb, PFC can carry more oxygen at equal concentration. The release of oxygen from PFC is realized by simple diffusion via the oxygen concentration gradient, while the release of oxygen from Hb is related to Hb oxy-deoxy conformational change. Thus, the efficiency of oxygen release from PFC may be higher than that of Hb. Oxygen-carrying nanomaterials have been used to increase oxygen concentration in tumor tissues and alleviate tumor hypoxia. However, it is difficult for the oxygen-carrying nanomaterials to thoroughly relieve the hypoxia because they would be subject to great losses in the blood circulation, with consideration of potential oxygen toxicity to organs if the initially carried oxygen is too high.

### Oxygen-generating nanomaterials for hypoxic tumor therapy

The concentration of oxygen in tumor tissues can also be increased by oxygen generated *in situ* in tumor sites. This would avoid any potential side effects of oxygen toxicity in the circulation. It has been recognized that H_2_O_2_ is overexpressed in tumor tissues because of abnormal metabolic processes. The catalytic decomposition of H_2_O_2_ in tumor sites is regional, accompanied by generation of oxygen which could freely diffuse for hypoxic tumor therapy. Nanomaterials that have been used to decompose H_2_O_2_ are mainly based on CAT and CAT-like nanozymes.

**Figure 3.**
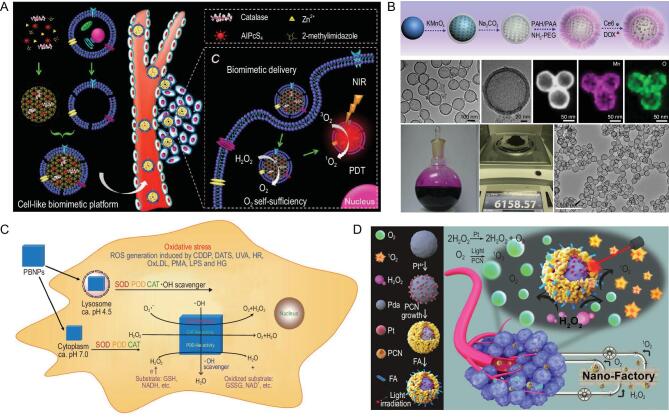
H_2_O_2_-based oxygen-generating nanomaterials for hypoxic tumor therapy. (A) CAT-loaded metal organic framework with cell-like biomimetic membrane for tumor-targeted PDT. Reproduced with permission from [70]. Copyright 2016, Wiley-VCH. (B) Synthesis of dual-drug loading H-MnO_2_-PEG nanoparticles and TEM images of H-MnO_2_-PEG. Reproduced with permission from [84]. Copyright 2017, Springer Nature. (C) Prussian blue nanoparticles with the ability of mimicking three antioxidant enzymes including CAT-like nanozymes to decompose H_2_O_2_ into oxygen. Reproduced with permission from [88]. Copyright 2016, American Chemical Society. (D) Preparation and functions of core-shell nanofactory for converting H_2_O_2_ from tumors into oxygen to enhance PDT. Reproduced with permission from [95]. Copyright 2018, Wiley-VCH.

Natural CAT super-efficiently catalyzes decomposition of H_2_O_2_ to generate oxygen. However, instability of CAT *in vivo* in the presence of some physiological proteases and poor half-life, limit the applications of CAT [68]. To overcome these shortcomings, research has focused on the renovation of CAT. Zhang's group loaded CAT into the pores of metal-organic frameworks (MOFs), which preserved the CAT from degradation [69,70]. With the generation of oxygen in tumor sites, the CAT-loaded MOFs encapsulated other drugs such as AlPcS_4_ to enhance PDT (Fig. 3A) and DOX to enhance chemotherapy. Polymers could be fabricated to encapsulate CAT with covalent conjugation. HA and fluorinated polyethyleneimine have both been used to load CAT and then accumulated in tumor tissues to generate oxygen for enhanced PDT or sonodynamic therapy (SDT) [71–73]. A CAT-loaded hydrogel was used to generate oxygen in tumor to overcome the immunosuppressive tumor microenvironment [74]. It was recognized that overcoming hypoxia would be helpful to enhance tumor radiotherapy. Tumor radiotherapy has been enhanced by CAT-loaded tantalum oxide (TaOX) nanoshells via two pathways: (i) generation of oxygen by decomposition of H_2_O_2_ to overcome hypoxia; (ii) concentration of radiation energy by high-Z element tantalum within the tumor to enhance radiation-induced DNA damage [75]. Natural CAT has high specificity and activity, but it has poor stability and is susceptible to the environment, especially complicated physiologic environments. Moreover, large-scale extraction or preparation of natural CAT is costly.

Besides natural enzymes, in 2013, nanomaterials with enzyme-like characteristics were defined as ‘nanozymes’ [76]. Development of these nanozymes has been rapid with the deeper understanding of nanotechnology and catalytic science. By mimicking or engineering the natural enzymes, nanozymes offer alternatives in many areas with advantages such as low cost, easy large-scale production, high stability and tunable activity. Among nanozymes, CAT-like nanozymes consisting of Prussian Blue (PB) or metals or metal oxides can efficiently catalyze decomposition of H_2_O_2_ into water and oxygen [77]. Next, we summarize that CAT-like nanozymes generate oxygen to alleviate tumor hypoxia for enhancement of tumor therapy.

Manganese ions play important roles in a variety of biological processes. The active sites of many enzymes contain manganese, such as manganese SOD, oxygen-evolving complex of photosystem II and CAT. In the last decade, the design and application of manganese oxidation catalysts have attracted keen interest in catalysis of H_2_O_2_ to generate oxygen. The form of nanomaterials containing manganese can be diversified with different constituents and structures. Manganese dioxide (MnO_2_) is the type most commonly modified for generation of oxygen [78]. Research has been done on hollow MnO_2_ nanoparticles, MnO_2_ nanodots and MnO_2_ nanosheets for decomposing H_2_O_2_ into oxygen for tumor therapy [79–83]. Not only could the hollow MnO_2_ catalyze the decomposition of H_2_O_2_ to alleviate the hypoxic condition, but it could also serve as a carrier to load high quantities of drugs with precisely controlled release because of its acid-responsive properties. The manganese ions released could be used in MRI to integrate diagnosis and treatment. The hollow MnO_2_ is uniform, with the potential for large-scale production for clinical use (Fig. 3B) [84]. The good performance of MnO_2_ means it is often combined with other materials to enhance the synergistic effects. MnO_2_ nanosheets can be anchored with calcinated TiO_2_-coated upconversion nanoparticles (UCNPs) to generate oxygen for circulated amplification of generation of ROS with 980 nm laser irradiation in deep hypoxic tumor tissue [85]. Metal ions and their complex compounds are often used as dopants to modify Mn-based nanomaterials. Mesoporous copper/manganese silicate nanospheres have been fabricated to enhance generation of ROS and GSH-activated Fenton reaction [86]. Manganese ferrite and ceria nanoparticle-doped mesoporous silica nanoparticles have been developed to scavenge ROS and produce oxygen for the polarization of macrophages [87]. Although many Mn-based nanoparticles have been developed, the synthesis of the nanoparticles is still at the lab, limiting their industrial translation. Furthermore, the high concentration of Mn^2+^ that would be released via the biodegradation of Mn-based nanoparticles may be toxic to cells and tissues. Therefore, sufficient data about their biocompatibility should be systemically investigated before further clinic translation.

PB, developed as a dye in the areas of paints and ink, is also used in medicine to detoxify patients from metal or elements poisoning. It has been approved as an antidote for thallotoxicosis by the FDA, illustrating its good biocompatibility for biomedical applications. Zhang's group first discovered that PB possessed multienzyme-like activity including CAT, peroxidase (POD) and SOD in 2016 (Fig. 3C) [88]. Since then, PB with CAT-like activity has been considered for oxygen generation in tumor therapy on the basis of excellent biologic security. PB nanoparticles were used as a core and coated with mesoporous organosilicon, which was loaded with photosensitizer [89,90]. H_2_O_2_ freely diffused into the inner PB, and was catalyzed into oxygen for the outer-loaded photosensitizer generating ROS to kill tumors. As a result of the high drug loading capability, hollow structure nanoparticles have attracted a lot of research interest. Hollow PB nanoparticles efficiently catalyzed H_2_O_2_ to generate oxygen, as well as loading drugs such as glucose oxidase for a cascade reaction to enhance starvation therapy [91]. Furthermore, hollow PB nanoparticles can generate heat energy under NIR light, offering an opportunity to combine photothermal therapy with enhanced starvation therapy. Although PB has good biocompatibility and has been used to catalyze the decomposition of H_2_O_2_ to generate oxygen_,_ its catalytic activity is not high in weakly acidic tumor environments.

Platinum (Pt)-based nanomaterials have drawn interest in applications of tumor diagnosis and treatment because of their near-infrared spectral absorption property and good biocompatibility. Pt nanoparticles possess both CAT-like and POD-like activity dependent on temperature and pH [92]. Many works have used Pt with diverse forms to generate oxygen for tumor therapy [93,94]. Zhang's group designed a hybrid core-shell nanoplatform, containing polydopamine cores, Pt nanoparticle interlay and porphyrin-based MOF shells, to ameliorate a hypoxic microenvironment and enhance generation of lethal ROS [95]. Thereby, the nanoplatform reduced invasion and metastasis of tumors (Fig. 3D). Liu's group designed a nanozyme based on Pt with dual enzyme-like activities (PtFe@Fe_3_O_4_) [96]. Under acidic conditions, the nanozyme exhibited both CAT-like activities and POD-like activities. PtFe@Fe_3_O_4_ could effectively overcome hypoxia and inhibit pancreatic cell growth. In addition to the dual enzyme activities with CAT-like and POD-like activities, Pt nanoparticles could also be combined with Au nanoparticles to construct CAT-like and glucose oxidase-like nanozyme [97]. The nanozyme shell with porphyrin-based MOF could be used for enhancement of synergistic tumor therapy with prevention of tumor metastasis and recurrence. Apart from Pt nanoparticles, a Pt complex, *cis, trans, cis*-[Pt(N_3_)_2_(OH)_2_(NH_3_)_2_], generated oxygen to alleviate hypoxia under light irradiation [98].

**Figure 4.**
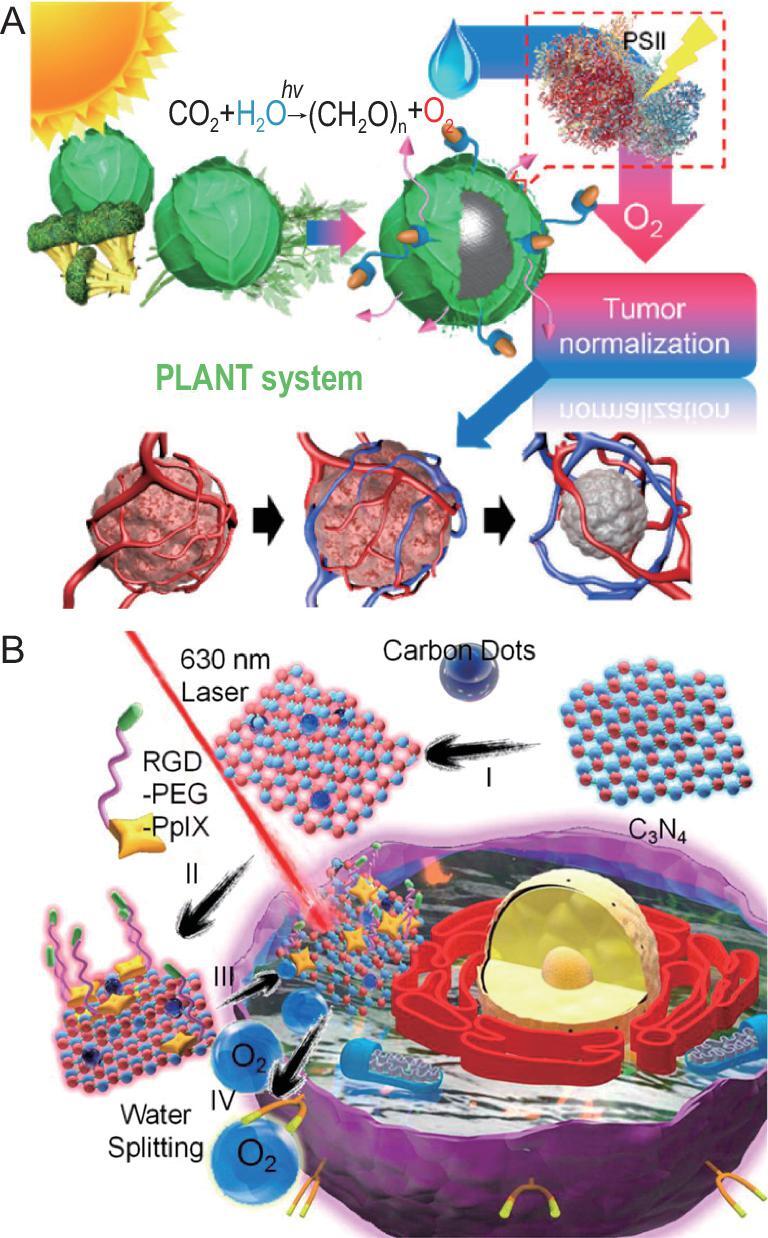
H_2_O-based oxygen-generating nanomaterials for hypoxic tumor therapy. (A) Tumor normalization by oxygen generation with thylakoid membrane-coated nanoparticles. Reproduced with permission from [103]. Copyright 2018, American Chemical Society. (B) Light-driven water splitting of nanocomposite for oxygen-generated sensitized PDT. Reproduced with permission from [104]. Copyright 2016, American Chemical Society.

Although these natural enzymes and nanozymes are able to catalyze H_2_O_2_ to generate oxygen in tumor sites for relieving tumor hypoxia, the amount of endogenous H_2_O_2_ in tumor tissues is restricted and it is difficult to generate abundant oxygen for efficient tumor therapy [99–101]. Liu's group developed a strategy to separately deliver H_2_O_2_ and CAT to tumor sites by liposomes. This strategy generated enough oxygen without the limitations of H_2_O_2_, which could not only enhance the therapeutic effects of radiotherapy but also help to reverse an immunosuppressive tumor microenvironment to favor antitumor immunity [102]. Although this strategy is novel and helps resolve the insufficient H_2_O_2_ at the tumor site, direct delivery of H_2_O_2_ in the blood circulation could lead to toxicity to organs because of its strong oxidizing property. Considering these problems, some researchers attempted to decompose H_2_O which is abundant at tumor sites in replacement of H_2_O_2_ for oxygen generation. In recent years, keen interest has been paid to water-splitting materials because of their potential applications in energy and environmental areas. Our group wondered about expanding the application of water-splitting materials to biomedical areas *in vivo*. Some natural materials (e.g. thylakoid) could be used to achieve efficient oxygen generation. Under 660 nm laser irradiation, the thylakoid membrane participated in the photosynthetic electron-transfer reaction to generate oxygen. Thylakoid membrane-coated synthetic nanoparticles normalized the hypoxic microenvironment and inhibited anaerobic respiration for hypoxic tumor treatment (Fig. 4A) [103]. However, extraction and preservation of thylakoid are complicated and the efficiency of oxygen generation is influenced by the physiologic environment. It is recognized that carbon nitride (C_3_N_4_, a water-splitting material) without the metal elements has potential for biomedical applications. Unfortunately, the absorption light of pure C_3_N_4_ is in the ultraviolet and visible range, which limits its biomedical applications because of low penetration depth with potential side effects of skin damage. Whereas the carbon dots were decorated into C_3_N_4_, the nanocomposite possessed enhanced red light absorption which could decompose H_2_O for oxygen generation [104]. With the addition of photosensitizer, the decorated C_3_N_4_ could overcome hypoxia and enhance the efficiency of PDT (Fig. 4B). Iron-doped C_3_N_4_ under two-photon irradiation has also been used to generate oxygen for tumor PDT [105]. Although C_3_N_4_ can be decorated and the absorption can be red-shifted, the modification is complex and time-consuming. Unlike C_3_N_4_, tungsten nitride (WN) with metallic property which can split water at a wavelength of 765 nm, is a promising material for oxygen production *in vivo*. WN has directly been used to provide oxygen via water-splitting for tumor oxygenation to treat tumor [106]. Water-splitting materials are often used for generating clean energy in the field of energy and environment, although the biocompatibility of these materials should be deliberated before clinical trial. Furthermore, high light energy is needed for these materials to split water into oxygen *in vivo*, which may lead to localized burning at the light irradiation site.

### Oxygen-economizing nanomaterials for hypoxic tumor therapy

**Figure 5.**
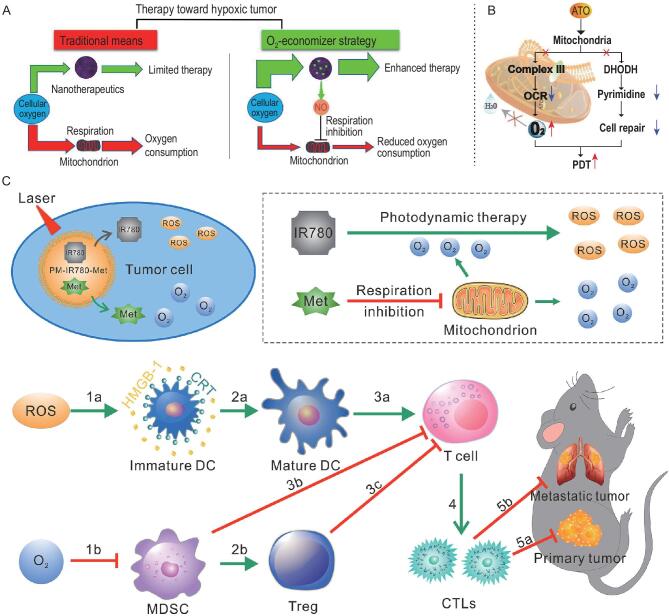
Oxygen-economizing nanomaterials for hypoxic tumor therapy. (A) Traditional therapeutic means and oxygen-economizer strategy for tumor treatments. Reproduced with permission from [109]. Copyright 2019, American Chemical Society. (B) Mechanism of enhancement of PDT by decreasing the cell oxygen consumption rate and improving the oxygen content by ATO. Reproduced with permission from [111]. Copyright 2019, Wiley-VCH. (C) Mechanism of enhancement of PDT and inhibiting mitochondrial respiration by IR780 and metformin to evoke immune response and inhibit primary tumor progression and tumor metastasis [116]. Reproduced with permission. Copyright 2020, Elsevier.

Respiration is the main mechanism that consumes oxygen in living cells [107]. Even though aerobic glycolysis is the major way for tumor cells to acquire energy, mitochondrial respiration still plays an important role in progression of tumors [108]. There has been research interest in the strategy of inhibiting respiration to economize oxygen for enhanced tumor therapy. Feng's group put forward the concept of an ‘oxygen-economizer’ to inhibit cell respiration and reduce oxygen consumption by relieving tumor hypoxia (Fig. 5A). The respiration was inhibited by the *in situ* generated NO in PLGA nanoparticles loaded with NO donor and Ce6, which could spare endogenous oxygen and further overcome the hypoxia barrier to enhance PDT [109]. Similarly, another group developed a method to reduce oxygen consumption by inhibiting mitochondria-associated oxidative phosphorylation with the oxygen-regulator atovaquone (ATO) (Fig. 5B). The ATO was self-assembled with PLGA-polyethylene glycol (PLGA-PEG) and photosensitizer verteporfin (VER) to form nanoparticles, which exhibited powerful antitumor PDT effects both *in vitro* and *in vivo* under laser irradiation [110]. ATO could also be encapsulated with indocyanine green derivatives in gelatin, which is sensitive for matrix metallopeptidase 2 (MMP-2) enzyme. This size-shrinkable gelatin-based vehicle stays intact before accumulating in tumor tissues, then the particle is transiently ruptured in the presence of MMP2 which is overexpressed in tumor tissues. This behavior has helped nanoparticles to enter deeply located, hypoxic regions to enhance tumor therapy [111]. Except for nanocarriers, ATO and a photosensitizer of Ce6-based self-delivery nanomedicine were designed via π−π stacking and hydrophobic interaction (ASCN). ACSN was carrier-free with a high drug loading rate and low excipient-induced systemic toxicity. The solubility and stability of ATO and Ce6 were dramatically improved by ACSN, which strengthened the cellular internalization and intratumoral permeability. The use of nanomedicine ACSN has been observed to result in tumor hypoxia relief and tumor growth inhibition [112]. As a potential alternative to ATO, metformin, commonly used as a first-line treatment for type II diabetes, can directly inhibit the activity of complex I in the mitochondrial electron transport chain. Thus, metformin was used to inhibit cell mitochondrial respiration and economize oxygen for tumor oxygenation [113]. Hydrophilic metformin and modified hydrophobic Ce6 were co-encapsulated in liposomes to modulate tumor hypoxia and enhance tumor PDT when the tumors were exposed to 660 nm laser light [114]. It was reported that metformin co-loaded with IR780 in amphipathic poly(ϵ-caprolactone)-poly(ethylene glycol)(PEG-PCL) was used to defeat relapsed and refractory malignancies by the synergistic effects of tumor oxygenation and PDT/PTT [115]. Furthermore, when the carriers were changed to longer circulation lifetime carriers of platelet membranes (PM), the alleviation of tumor hypoxia by inhibition of mitochondrial respiration with metformin could weaken the myeloid derived suppressor cell (MDSC)-regulated immunosuppressive pathway, and the oxygen-boosted PDT could trigger the immunogenic cell death (ICD)-based pathway (Fig. 5C). Finally, the activated immune system inhibited tumor metastasis [116]. These results showed that inhibiting mitochondrial respiration effectively oxygenated tumor and confirmed good therapeutic effects for tumor inhibition when combined with other therapeutics. Further ways to modulate cell metabolism and economize oxygen by reducing oxygen consumption in tumors should be explored. A novel strategy to supplement oxygen by reducing oxygen consumption, as well as many misgivings including the efficiency of hypoxia alleviation and the side effects of inhibiting mitochondria respiration, is emerging.

## DIMINISHING OXYGEN DEPENDENCE OF NANOMATERIALS FOR HYPOXIC TUMOR THERAPY

Oxygen supply at tumor sites could improve oxygen-dependent tumor therapy; however, the increased oxygen at tumor sites is restive. If the increase in oxygen is insufficient, the promotion of oxygen-dependent tumor therapy is limited; on the contrary, the increased oxygen in the host may lead to side effects. For instance, it is recognized that hyperbaric oxygen therapy may cause diseases of hyperoxic seizures or barotrauma sickness [117]. Therefore, oxygen-independent tumor therapies are urgently required to treat hypoxic tumors.

### Therapeutic gas-generating nanomaterials for hypoxic tumor therapy

Some gaseous molecules play an essential role in physiological modulation. Gas therapy, a promising therapeutic strategy, is under development for treatment of inflammation-associated diseases. Among these, distinguished therapeutic effects of NO on cardiovascular diseases generated a Nobel Prize in Physiology or Medicine in 1998. Gaseous molecules can freely diffuse into deep tumor tissue. As a consequence, gas therapy has potential for treatment of hypoxic tumors because of it great penetrability. As gas is apt to aimlessly diffuse throughout the whole body, approaches are required for its delivery in a controllable concentration and specific accumulation in tumor tissues to reduce any potential systemic side effects. To date, gas therapy treatments have mainly involved inhalation of gas, oral administration or gas prodrugs. However, both the inhalation and oral administration of gas are impeded by non-specific delivery and off-target toxicities. Strategies using gas prodrugs or nanomedicines offer a potential solution for gas-controllable delivery in biomedical applications.

**Figure 6.**
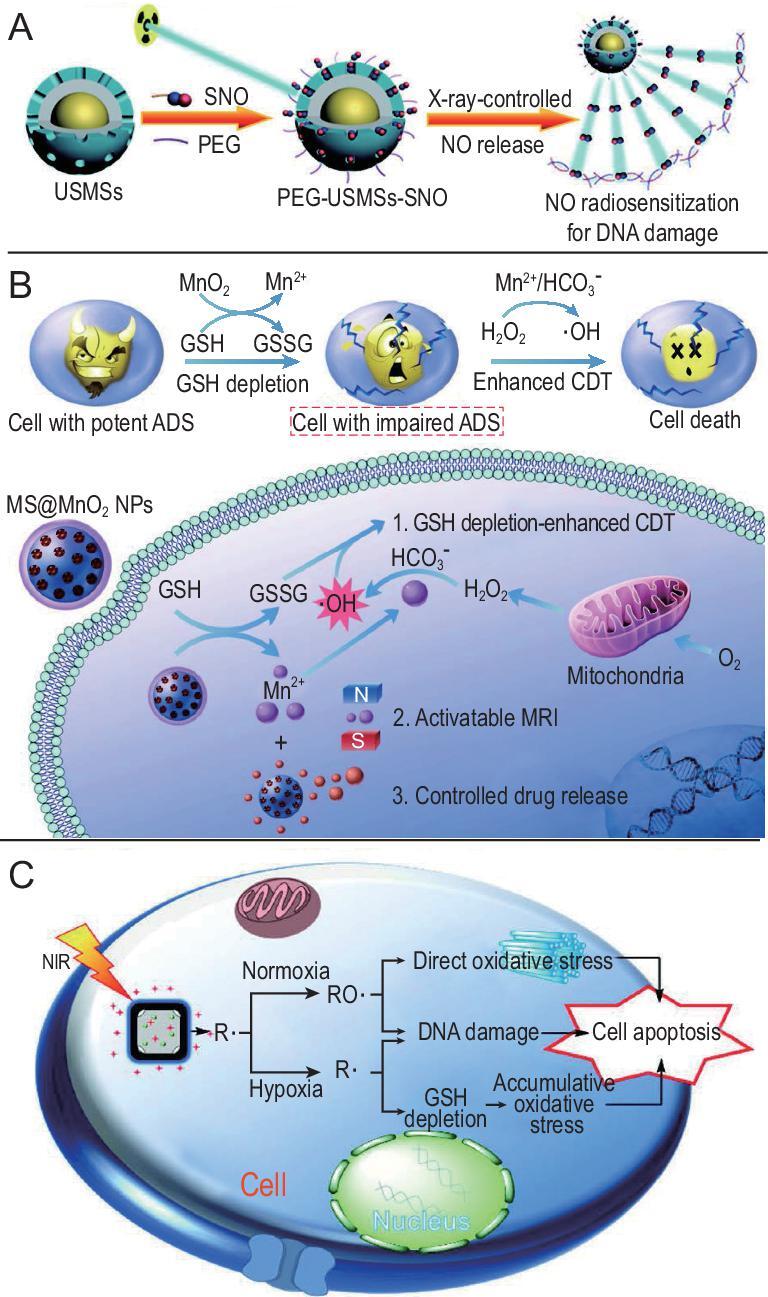
Diminishing oxygen dependence of nanomaterials for hypoxic tumor therapy. (A) X-ray controlled NO release to sensitize radiotherapy for DNA damage. Reproduced with permission from [126]. Copyright 2015, Wiley-VCH. (B) The mechanism of MnO_2_ as a smart chemodynamic agent for enhanced CDT of tumors. Reproduced with permission from [141]. Copyright 2017, Wiley-VCH. (C) Therapeutic mechanism of the free radicals under different oxygen tensions. Reproduced with permission from [146]. Copyright 2017, Wiley-VCH.

NO, a messenger molecule, has essential roles in several physiological functions [118,119], including the immune response, angiogenesis and cardiovascular homeostasis. However, NO is a concentration-dependent ‘double-edged sword’ in tumor therapy. At high concentration (>1 mM), NO directly kills tumor cells for tumor inhibition. At relatively low concentration (1 μM−1 mM), NO not only inhibits expression of P-glycoprotein and multidrug resistance-associated proteins [120], but also relieves tumor hypoxia, which offers opportunities for synergistic tumor therapy [121]. The mechanisms of NO to relieve hypoxic tumor include modulating blood vessel relaxation to increase blood flow, accelerating the metabolism of intracellular GSH [122] and decreasing tumor oxygen consumption rate to oxygenate tumor [123]. The great effects of NO on alleviation of hypoxia are pushing strategies to achieve targeted delivery and controllable release of NO in tumor tissues in hypoxic microenvironments to be developed for subsequent synergistic therapy [124]. Sortino's group has done lots of work in this area involving NO release in nanoassemblies [125]. To treat hypoxic tumor, Shi's group designed an X-ray-activated synergistic NO gas/radiotherapy system (PEG-USMSs-SNO) via engineering UCNPs with S-nitrosothiol (R-SNO)-grafted mesoporous silica. NO was specifically released after the breakage of S-N bond under X-ray radiation, then the released NO could stimulate hypoxic tumor radiosensitization and further improve the radiotherapeutic effects against hypoxic tumors (Fig. 6A) [126]. Besides its sensitization functions, NO has been reported to treat deeply hypoxic tumor because of its free diffusing functions. An NO generator was designed by oxidation of l-arginine, which was incorporated into a porphyrinic metal-organic framework (l-Arg@PCN). The l-Arg@PCN was subsequently coated with homologous targeting tumor cell membrane to accumulate in tumor tissues. Local generation of ROS by PCN under NIR irradiation led to the converse, l-Arg into NO. By combining PDT and gas therapy, an increase in oxidative/nitrification stress and inhibition of growth and proliferation capability of tumor cells were realized [127]. Gaseous molecules regulate some physiological processes, but the regulatory effects are transient and limited. Combining gas therapy with other therapeutic approaches can yield better therapeutic performance.

### Radical-generating nanomaterials for hypoxic tumor therapy

Free radicals exist within living organisms to maintain homeostasis. Almost all free radicals are highly reactive because of their unpaired valence electrons. They play important roles in cell metabolism in the normal level, but at abnormally high levels they can randomly destruct cellular ingredients (lipid membrane, proteins and DNA) and cause cell death. Unlike type II PDT (singlet oxygen generation based on energy transfer from excited photosensitizers to oxygen molecules) generating singlet oxygen dependent on oxygen and only initiated in a well-oxygenated environment, free radicals such as hydroxyl radicals (·OH) and carbon-centered radicals (R·) can be produced in the absence of oxygen [128]. Because of their high reactivity and oxygen-independent generation ability, free radicals have potential hypoxic tumor therapy.

In contrast to the type II PDT used in most existing PDT systems, type I PDT can transfer energy from photosensitizers to other substrates (e.g. H_2_O) and produce respective free radicals. Many studies have reported that type I PDT has great efficiency, even under hypoxic conditions [129,130]. Titanium dioxide (TiO_2_) is widely used in nanomaterials, with high photocatalytic activity and good biocompatibility. When TiO_2_ nanomaterials absorb suitable photon energy (generally ultraviolet), the photogenerated holes can oxidize the surrounding H_2_O to form ·OH [131]. A series of studies on the killing effects of TiO_2_ on different tumor cells was carried out *in vitro* under ultraviolet light irradiation. Subsequent animal experiments demonstrated the good antitumor effect of TiO_2_, with the skin at tumor sites cut open for irradiation because of the limits of penetration of ultraviolet [132]. Attempts were made to prolong the activated wavelength of TiO_2_ for better antitumor effects *in vivo*. In one instance, a carbon-nanodot-decorated TiO_2_ nanotube composite was developed as carbon-nanodot can transfer light from 650 nm to 325–425 nm, which prolonged the activated wavelength to produce ·OH [133]. UCNP (e.g. NaYF4: Yb^3+^, Tm^3+^)-decorated TiO_2_ have been developed via near-infrared-to-ultraviolet optical conversion for near-infrared light triggering photochemical reaction of TiO_2_ [134]. Hydrogenated TiO_2_ demonstrated efficient optical absorption of near-infrared light, which is beneficial for direct excitation of TiO_2_ with near-infrared irradiation [135]. However, there are still some challenges to clinical applications of TiO_2_, such as the efficiency of type II PDT and the biocompatibility.

Some metal irons (e.g. Fe^2+^, Mn^2+^) can transform H_2_O_2_ into ·OH without the help of oxygen and external energy input, according to Fenton or Fenton-like reactions [136]. Because ·OH is able to oxidize most organic molecules including lipids, proteins and DNA at high rates, Fenton or Fenton-like reactions can be used to kill tumor cells independently of oxygen [137]. Bu's group first suggested the concept of chemodynamic therapy (CDT), which used Fenton or Fenton-like reactions to generate ·OH and induce cell death independent of oxygen concentration and without the need for external energy input by laser irradiation [138]. PEGylated single-atom Fe-containing nanocatalysts have been reported that can effectively generate ·OH in an acidic tumor microenvironment [139,140]. Chen's group designed a self-reinforcing CDT nanoagent based on MnO_2_, which depleted intracellular reductive glutathione, and the generated Mn^2+^ induced a Fenton-like reaction to destroy tumor cells [141]. Specifically, in the presence of bicarbonate (HCO_3_^−^), MnO_2_ reacted with glutathione to yield glutathione disulfide and Mn^2+^, which could trigger ·OH production from H_2_O_2_ with the help of HCO_3_^−^ (Fig. 6B). A porous MIL-100(Fe) layer was coated on Fe_3_O_4_ nanoparticles, followed by attachment of upconversion nanoparticles (Fe_3_O_4_@MIL-100(Fe)-UCNPs, or FMU) for hypoxic tumor therapy. UCNPs could transfer NIR to UV/vis light, and Fe_3_O_4_@MIL-100(Fe) absorbed UV/vis light to generate ·OH independent of tumor oxygen. Based on a photo-Fenton reaction, FMU could produce more ·OH for hypoxic tumor therapy [142]. Similar to the above mentioned problem of the limited content of endogenous H_2_O_2_, the amounts of endogenous H_2_O_2_ also limit the catalytic efficacy of the Fenton reaction. Zhang's group developed an ATP-responsive Fenton nanosystem by autocatalyzing Fe^3+^ into Fe^2+^ as well as the generation of H_2_O_2_ under glucose oxidase to produce ·OH. The nanosystem generated adequate H_2_O_2_ by glucose oxidase catalyzing glucose and then induced remarkable production of ·OH by the Fenton reaction [100]. Vitamin C-loaded mesoporous magnetic nanocubes (MMNCs) with ROS self-generation and self-enhancement also have been used for hypoxic tumor therapy. Vitamin C served as an original source for H_2_O_2_ generation and MMNCs possessed Fenton reagent-like activity for ROS self-enhancement to treat hypoxic tumor [143]. Our group developed a Fenton-like bioreactor based on engineered facultative anaerobes for tumor therapy. Facultative anaerobes choose to travel to the tumor site because of the hypoxic tumor microenvironment, which could be used to transport nanomedicine to tumor regions. The bacteria overexpressed respiratory chain enzyme II to increase H_2_O_2_ generation, which was beneficial to realize a self-supplied therapeutic Fenton-like reaction [144].

Other than ·OH, R· are generated in the absence of oxygen and high toxicity to cells. Thermally decomposable azo initiators are commonly used in generation of R·, and are always applied in free radical polymerization and biological systems to produce oxidative stress [145]. Generation of radicals by decomposing these initiators is thermally dependent, and thus is limited under physiological conditions. A heater is required to speed up production of free radicals for tumor therapy. Almost simultaneously, Xia's group and Zhang's group designed similar systems with initiator loaded-gold nanocages, which were filled with a phase-change material to realize the controllable production of free radicals for tumor therapy [146,147]. Gold nanocages, photothermal conversion agents with fine biocompatibility, were used both as heat source and initiator carrier. The radical source came from the same initiator, 2,2^′^-azobis[2-(2-imidazolin-2-yl)propane] dihydrochloride (AIPH). Under NIR irradiation, the produced heat by gold nanocages not only caused decomposition of AIPH to generate R·, but also released the blocked R· with the phase transition of the copolymer. This free radical-based therapy had good antitumor efficacy regardless of whether oxygen was sufficient or not (Fig. 6B). To expand the multifunctional or theranostic applications in hypoxic tumor therapy, it is essential to monitor R· release in real time. A smart free-radical system was developed to monitor production of free radicals in real time. Fluorescent dyes and quenchers were linked with the initiators, which could homolytically cleave in the presence of free radicals. After homolytic cleavage of the initiators, the fluorescent dyes and quenchers were separated and recovered because of the R· production [148]. Although promising, this approach to generate R· for hypoxic tumor therapy requires improvement in some respects before clinical application, such as the potential cytotoxicity from unsatisfactory degradability of inorganic photothermal agents, low initiator loading capacity and limited generation efficiency of R· for hypoxic tumor therapy.

## CONCLUSION AND PERSPECTIVES

Recent studies of different strategies to treat hypoxic tumors based on nanomaterials were categorized as (i) elevating oxygen level via nanomaterials in tumor tissue for enhancement of oxygen-dependent tumor therapies and (ii) therapies with diminishing oxygen dependence of nanomaterials. Tumor hypoxia could be relieved by supplying oxygen in tumors through use of oxygen-carrying nanomaterials, oxygen-generating nanomaterials or oxygen-economizing nanomaterials, which sensitized or enhanced therapeutic effects of oxygen-dependent tumor therapy. Because of the complexity of hypoxic tumors, much research has also focused on strategies with diminished oxygen dependence of nanomaterials for hypoxic tumor therapy.

Although lots of exciting results have been put forward over the past decade, challenges remain for clinical applications of nanomaterials for treatment of hypoxic tumors, including biocompatibility and validity of nanomaterials. One of the most critical concerns regarding nanomaterials is the biocompatibility, particularly in nanomaterials containing heavy metals, and nanomaterials with good biocompatibility should be preferentially selected. Similar to implantable nanomaterials, biocompatibility can be improved by surface modification, including chemical modification and physical modification. In addition, because of the dynamic changeable hypoxic conditions, it can be hard to continually alleviate hypoxia in tumor therapy through use of oxygen-carrying nanomaterials or oxygen-generating nanomaterials. Premature release of oxygen from oxygen-carrying materials can occur in the blood circulation, which may lead to cytotoxicity to other host organs. Thus, there is a need to develop nanomaterials subject to controllable release of oxygen rather than simply being oxygen carriers. Fortunately, there have been reports of controllable release of drug from nanomaterials via exogenous or endogenous stimulation, so these approaches may be applicable to controllable release of oxygen from oxygen-carrying nanomaterials. The water-splitting nanomaterials used to generate oxygen also face several problems including low efficiency, poor dispersion and unknown biocompatibility *in vivo*. Development of water-splitting nanomaterials seems to have hit a ‘bottleneck’, but we believe the related problems will be solved in future. Furthermore, therapies based on nanomaterials with diminished oxygen dependence could directly inhibit growth of tumors, with the potential to avoid the concerns regarding oxygen-related nanomaterials. But specific killing of tumor cells by those nanomaterials with diminished oxygen dependence requires further attention. Targeting ability of such materials may be realized by modifying materials with suitable targeting groups or targeted cell membrane. Furthermore, many nanomaterials used to treat hypoxic tumors are dependent on laser irradiation, but the penetration depth of the used laser in the body is insufficient. Thus, strategies are required for treatment of hypoxic tumors based on nanomaterials without limitation in penetration depth of laser.

There has been much development in immunotherapies in recent years, but the therapeutic effects of immunotherapy can also be limited by hypoxic conditions, e.g. expression of HIF-1α in the tumor microenvironment. Nanomaterials designed to modulate hypoxia and improve tumor immune microenvironment such as immune cells may be explored in future. In all, it is believed that nanomaterials are increasingly important in the field of hypoxic tumor therapy as a result of their great drug delivery, good tumor accumulation and various functions.
